# Vision-Related Quality of Life and Seasonal Affective Disorder in Patients with Glaucoma and Macular Degeneration

**DOI:** 10.3390/vision6020032

**Published:** 2022-06-14

**Authors:** Kira J. Szulborski, Miranda D. Prosniewski, Sidrah Anjum, Amer Mosa Alwreikat, Patrick R. Aquino, David J. Ramsey

**Affiliations:** 1 Department of Surgery, Division of Ophthalmology, Lahey Hospital & Medical Center, Beth Israel Lahey Health, Burlington, MA 01805, USA; kira.szulborski@tufts.edu (K.J.S.); miranda.d.prosniewski@lahey.org (M.D.P.); sidrahanjum1990@gmail.com (S.A.); amer.alwreikat@lahey.org (A.M.A.); 2 Department of Ophthalmology, Tufts University School of Medicine, Boston, MA 02111, USA; 3 Department of Psychiatry & Behavioral Medicine, Lahey Hospital & Medical Center, Beth Israel Lahey Health, Burlington, MA 01805, USA; patrick.aquino@lahey.org; 4 Department of Psychiatry, Tufts University School of Medicine, Boston, MA 02111, USA

**Keywords:** seasonal affective disorder, moderate vision loss, vision-related quality of life, macular degeneration, glaucoma, visual function questionnaire, seasonal pattern assessment questionnaire

## Abstract

Seasonal affective disorder (SAD) is characterized by depressive episodes related to changes in the seasons. Patients with severe vision loss are at an increased risk of SAD. This study seeks to determine the extent to which patients with moderate vision loss report symptoms of SAD. In this cross-sectional, comparative case series, the Seasonal Pattern Assessment Questionnaire (SPAQ) and the National Eye Institute Visual Function Questionnaire (VFQ-39) were used to screen 111 patients with age-related macular degeneration (AMD) and/or primary open-angle glaucoma (POAG). A multiple regression analysis was performed to create a predictive model for SAD based on the Global Seasonality Score (GSS) using the VFQ-39. Subjects who reported symptoms of SAD (GSS > 8) had lower vision-related quality of life (composite score: 57.2 versus 73.2, *p* < 0.001). Exploratory factor analysis revealed that the items on the VFQ-39 split into two distinct dimensions that together accounted for 63.2% of the total variance in the GSS. One group of questions addressed vision-related problems; the other group comprised questions related to the quality of life. Whereas this model successfully identified patients with vision loss at risk of SAD, a model restricted to the questions available on the shorter, widely used VFQ-25 instrument did not reliably identify patients at risk of SAD.

## 1. Introduction

Seasonal affective disorder (SAD) is characterized by recurrent episodes of major depression, mania, or hypomania that regularly occur in relation to the seasons. Many patients with depression have a SAD component, especially during the winter months when there is less daylight and the nights are longer. SAD affects an estimated 5% of the United States population—more than 14.5 million Americans [1]. It is significantly more common among individuals living at northern latitudes, with nearly 10% of the population in the northeastern United States affected by SAD [2]. Some individuals experience symptoms of SAD on top of existing major depression, but others have subsyndromal SAD (sub-SAD), a milder form having symptoms of lowered mood in the wintertime but not meeting the criteria for clinical depression.

Patients with vision loss have, in general, a higher incidence of depressed mood, anxiety, and sleep disturbances compared with the general population [3,4,5,6]. A recent study of patients recruited from a survey of the Danish Association of the Blind registrants whose self-reported vision was limited to light perception (severe vision loss) had an increased incidence of SAD [7]. However, to date, no study has examined the incidence of SAD among patients who have specific ophthalmic disorders that typically result in moderate vision loss. The aim of this study is to determine the extent to which patients with vision loss from age-related macular degeneration (AMD) and/or primary open-angle glaucoma (POAG) experience symptoms related to SAD and to determine whether this symptom score can be predicted by commonly used measures of vision-related quality of life.

## 2. Materials and Methods

The study constituted a cross-sectional comparative case series of patients who were seen by the ophthalmology service at Lahey Hospital & Medical Center diagnosed with intermediate to advanced AMD and/or moderate to advanced POAG. The research followed the tenets of the Declaration of Helsinki and was approved by the Institutional Review Board of the Lahey Hospital & Medical Center in Burlington, Massachusetts. Subjects were identified based on ICD-10 diagnosis codes and recruited between March 2017 and April 2019. Each prospective participant was sent a letter of invitation asking them to participate in the study by completing an enrollment form and filling out a Seasonal Pattern Assessment Questionnaire (SPAQ) [8,9], and a National Eye Institute (NEI) Visual Function Questionnaire 39 (VFQ-39) [10]. A pre-paid business reply mail envelope was enclosed with the materials. Written informed consent was obtained from all patients enrolled in the study. No patients in the study experienced any adverse events. Patients were encouraged to share any concerns raised by the survey instruments with their primary care or eye care providers.

A detailed chart review was conducted for all patients enrolled in the study by a retina (DJR) and glaucoma (AA) specialist to confirm the presence and severity of AMD and/or POAG, as well as to identify other ocular conditions that might cause visual disability. Demographic and clinical data related to ocular health were extracted from each patient’s chart. These included date of birth, sex, self-reported race or ethnicity, history of ophthalmic diseases, history of major depression or the use of antidepressant medications, visual acuity (VA), cup-to-disk ratio (CDR), Humphrey visual field test results, and lens status. Patients with low vision status were defined as having a VA in the better eye of worse than 20/70 [11]. Classification of patients who reported symptoms of SAD was based on a Global Seasonality Score (GSS) of greater than eight on the SPAQ.

Data were coded in Microsoft Excel 2010 (version 14.0, Microsoft Corporation, Redmond, WA, USA) and analyzed using RStudio Version 1.1.422 (RStudio: Integrated Development for R. RStudio, PBC, Boston, MA, USA) with the missMDA package [12] to impute missing data for analysis and SPSS Statistics software version 22.0 (IBM Corp, Armonk, NY, USA). All tests were 2-sided, and *p*-values below 0.05 were regarded as statistically significant. A principal component analysis (PCA) with varimax rotation was performed on the VFQ-39 survey items with Cronbach’s α to assess internal consistency reliability. Linear regression analysis was conducted to assess the relationship between the GSS from the SPAQ and various parameters in patients with AMD and POAG, as well as questions from the VFQ-39 and 25-Item Vision Function Questionnaire (VFQ-25), a subset of the VFQ-39. Stepwise multiple regression analysis was performed to create a predictive subscale for screening SAD/sub-SAD in patients. A receiver operator characteristic (ROC) curve was constructed by plotting sensitivity against 1-specificity, calculated for each value observed.

## 3. Results

A total of 350 patients were identified who met the criteria for inclusion in the study. Study packets that included the SPAQ and VFQ-39 were returned by 114 subjects (32%). Three of these participants were excluded because of incomplete surveys. All participants identified English as their primary language and completed both surveys in English. The return rate was greater for subjects with AMD (36%) compared to POAG (26%) (*p* = 0.027), despite the former group having worse VA (*p* < 0.001).

The demographics and clinical characteristics of the 111 study participants are summarized in Table 1. At the time of the survey’s completion, patients ranged in age from 60 to 98 years old (mean 81.2 ± 8.9 years), 59% were female, and nearly all participants self-reported their race as white (99.1%). A total of nine patients (8.1%) had a history of depression, and three patients (2.7%) were recorded as taking antidepressant medications. Patients with POAG had an average mean deviation (MD) of −8.01 ± 7.00 decibels on the Humphrey 24-2 visual field. Seven patients had unreliable visual fields, and three were unable to perform visual field testing because of low vision.

From the participants included in the subgroup analysis of patients classified as having POAG or AMD, it was necessary to remove seven patients who were found upon a detailed review of the medical record to have both AMD and POAG, as well as seven patients with POAG who also had other conditions that likely contributed to vision loss (four patients with retinal vein occlusions in one eye, and one patient each with vision loss attributable to diabetic retinopathy, corneal scarring, and traumatic vision loss in one eye). The characteristics of the patients with POAG and AMD were similar with regard to GSS, age, history of depression, and measures of vision-related quality of life.

### 3.1. SAD and Sub-SAD Classification

The GSS, as determined by each subject’s SPAQ responses, was used to assess whether they demonstrated symptoms of SAD. This measure reflects sleep length, social activity, mood, weight, appetite, and energy level as reported by the subjects on the SPAQ. A GSS of greater than 11 is considered indicative of SAD, whereas scores of 9 or 10 are categorized as sub-SAD. Following these guidelines, 18.9% of patients were identified as having symptoms of SAD or sub-SAD (16 patients at risk for SAD and 5 for sub-SAD), while the remaining 90 patients (81.1%) fell below the threshold associated with risk of the condition. At the time of the administration of the survey, the group of patients with a GSS likely indicated SAD/sub-SAD were significantly older than those below this threshold (84.7 ± 5.6 years versus 80.3 ± 9.4 years, *p* = 0.008). Univariate analysis demonstrated that GSS scores showed no correlation with age, VA, sex, history of depression, season (based on the date of survey completion), lens status, low vision status, or type of vision disorder (AMD vs. POAG).

### 3.2. VFQ-39 Responses

Patients meeting the SAD/sub-SAD criteria reported lower vision-related quality of life as reflected in lower VFQ-39 composite scores as compared with those without SAD (57.2 ± 21.1 points vs. 73.2 ± 17.9 points, *p* < 0.001). Principal components analysis (PCA) was performed for the VFQ-39 survey items where the factor loadings showed strong correlations (r > 0.60) [13]. This identified two distinct factors that account for 63.2% of the total variance in the GSS. The first component encompassed vision-associated problems, while the second captured measurements pertaining to the quality of life (Table 2; Supplemental Table S1). This provides evidence for construct validity in the VFQ-39 survey. Cronbach’s α calculated for this set of items on the VFQ-39 survey gave a high internal consistency reliability of 0.933.

Item-level correlations were performed to determine what questions predicted whether a patient fell into the SAD/sub-SAD group. Based on their correlations with GSS, items 10, 11, 13, 15c, A5, A8, and A9 were shown to possess predictive ability for SAD/sub-SAD (r > 0.60, *p* < 0.001).

Stepwise regression was performed to assess the ability of items in the VFQ-39 to predict the risk of SAD/sub-SAD in all patients (*n* = 111), including those who had both AMD and POAG. The resulting model included items 4, 6, 7, 10, A1, A5, A7, and A8. The final model showed an R^2^ of 0.541 (*p* < 0.001). Subsequent hierarchical regression modeling of the variables selected by stepwise regression analysis resulted in a subscale model containing items 4, 7, 10, A1, A5, A7, and A8, and was shown to be the best inclusive model (R^2^ = 0.483, *p* < 0.001). Cronbach’s α calculated for these seven items gave an internal consistency reliability of 0.830. Further reliability analysis revealed that dropping item 4 raised the internal construct reliability to 0.860. Based on these results, question 4 was excluded from the final model.

### 3.3. Receiver Operator Characteristic (ROC) Curve Analysis

The diagnostic performance of our model was evaluated using ROC curve analysis to analyze the capacity of our model to predict the risk of SAD/sub-SAD. The ROC curve was used to identify the best cutoff point that maximized sensitivity and specificity in discriminating patients based on their GSS score. A model using all questions from the VFQ-39 demonstrated remarkably high internal reliability and consistency with a Cronbach’s α of 0.860, allowing for the creation of a useful classification model. By contrast, restricting a model to only those questions on the VFQ-25 was inadequate for classifying patients based on their GSS score (Cronbach’s α = 0.30). The area under the curve (AUC) for our final subscale model of questions on the VFQ-39 was calculated to be 0.955 (95% CI: 0.916, 0.994; Supplemental Figure S1). Additionally, this model demonstrated a sensitivity of 95.5% (95% CI: 86.8%, 104.2%), specificity of 86.6% (95% CI: 78.4%, 94.7%), a positive predictive value of 70% (95% CI: 53.6%, 86.4%), a negative predictive value of 98.3% (95% CI: 95%, 101.6%), and an overall accuracy of 88.8% (95% CI: 88.5%, 89.0%). In applied psychology of behavior, with all the factors that can influence human behavior, an AUC value of 0.70 or higher is considered a strong effect [14].

## 4. Discussion

Our study identified a surprisingly large number of patients with moderate visual impairment owing to AMD or POAG who screened positive with a validated instrument for risk of SAD (18.9%). This is even more surprising because our study population was composed of predominantly older adults. The prevalence of SAD across the lifespan has suggested that it is typically more common in younger adults than in older adults [2,15]. Though widely used in research, the SPAQ may best operate as a screening instrument. Future studies into the prevalence of SAD among patients with AMD and POAG should seek to confirm the SAD diagnosis by using clinically-validated diagnostic instruments, such as the Hamilton Depression Rating Scale—Seasonal Affective Disorder Version Self Rating Version [16]. Even if only half of the patients identified are confirmed to have SAD, this would still be higher than the expected rate for our population of older adults in northern latitudes.

This study also indicates that the NEI VFQ-39 may allow for the identification of SAD/depression in patients with vision loss from AMD and POAG. However, many of the questions that are most closely associated with a risk of SAD/sub-SAD are not present on the shorter, more widely used VFQ-25. The NEI VFQ-25 questionnaire comprises 26 questions, which are classified into the following 12 subscales: general health (1 item); general vision (1 item); ocular pain (2 items); difficulty with near-vision activities (3 items); difficulty with distance-vision activities (3 items); limitation of social functioning due to vision (2 items); mental health problems due to vision (4 items), role limitations due to vision (2 items); dependency on others due to vision (3 items); driving difficulties (3 items); difficulty with color vision (1 item); difficulty with peripheral vision (1 item). The VFQ-25 instrument is the product of dimension reduction of the NEI’s original 51-item VFQ [17], developed to assess health-related quality of life in patients with visual impairment and its relationship to vision-related function. The VFQ-25 has an optional appendix of 13 additional items taken from the original 51-item version, bringing the total to 39 questions per survey to comprise the VFQ-39, an instrument confirmed to have comparable validity [10,18]. Although the VFQ-25 possesses good psychometric properties and can provide reliable and valid data on visual functioning in patients with ophthalmic problems, including those with AMD [19] and POAG [20], it loses dimensionality resulting from the additional questions missing from each subscale. The inclusion of the appendix questions can, therefore, be helpful for a specific condition under study [21].

In this study examining risk for SAD, a subtype of depression known to affect patients with vision-impairing conditions [22], a model restricted to questions present on the VFQ-25 version failed to achieve a sufficiently high internal reliability and consistency to classify the patients for risk of SAD based upon their GSS score. The current mental health subscale of the VFQ-25 (items 3, 21, 22, and 25) primarily considers social functioning and measures anxiety related to visual impairment more than depression. This shows that while the more commonly used VFQ-25 instrument is inadequate for capturing the risk of SAD/sub-SAD, the expanded version of the instrument does a much better job, and it requires only a subset of the supplemental questions to do so. We propose that the addition of an abbreviated appendix consisting of a subset of validated questions from the more comprehensive VFQ-39 to the VFQ-25 could allow for an efficient and accurate way to identify patients with SAD or depression. We would anticipate that subsequent studies would utilize clinical assessment to identify cases of SAD to further refine this instrument. Furthermore, the utility of identifying patients at risk for SAD with a commonly used ophthalmologic assessment instrument could decrease the barriers to the diagnosis and/or treatment of SAD in real-world applications.

SAD, similar to major depression, is a mood disorder that often requires long-term management. It can increase the risk of visual impairment from conditions such as AMD and POAG by lessening the motivation of patients to complete the monitoring of these diseases or to accept and adhere to necessary treatments [23,24,25,26,27]. Studies have shown that subjective depressive symptoms and a clinically-diagnosed depressive disorder independently and synergistically increase the risk of the incidence or progression of glaucoma [28,29] and the rate of vision loss from AMD [30]. Retinal dysfunction has also been hypothesized to play a role in the pathogenesis of SAD, specifically from the loss of intrinsically photosensitive retinal ganglion cells (ipRGCs) [7]. Although our study did not find a difference in the prevalence of SAD in patients with AMD compared to those with POAG, it is very likely that visual pathways do play an important role in the development and course of this mood disorder.

Psychotherapy and medications are common treatments for patients with SAD, but light therapy (phototherapy) is another form of treatment that may be utilized alone or in conjunction with other forms of treatment. Narrow-band blue-light treatment has been suggested to be equally as effective as bright white-light treatment [31,32]. The discovery of ipRGCs with their novel photoreceptor pigment, melanopsin, which is sensitive to blue light (470–490 nm), has led to research on the effects of short-wavelength light on human behavior [33]. Patients who have POAG and experience subsequent loss of retinal ganglion cells, including the subset of ipRGCs, may be more susceptible to developing SAD compared with patients who have AMD, which primarily impacts the central retinal function [34,35].

This study is limited to the evaluation of a relatively small group of patients sampled from a single academic ophthalmology center in a northern latitude. The study group, though representative of the population served by our medical center, lacked diversity and may, therefore, not represent the extent of SAD among patients of other races or ethnicities with AMD and/or POAG. Additionally, the study spanned many different months. Although some evidence has shown that the month of completion of a survey has no influence on SAD criteria [36], timing the taking of surveys to periods of the year where SAD is more commonly experienced might better capture how symptomatic patients become during the winter months. The SPAQ shows a good specificity (94%), but a low sensitivity (44%) [37], and depending on the cut-off score used for the GSS, reported sensitivity has ranged from 40% to 94% [38]. While the SPAQ has high sensitivity and specificity for identifying individuals with SAD/sub-SAD compared with those without these conditions, it performs poorly for differentiating between SAD and sub-SAD [39]. Our pilot study is also not designed to allow us to compare the individual subscales of the VFQ-39 instrument between our different study groups. A larger study is necessary to overcome this limitation. Finally, although we account for variations in lens status among the participants in our study, we lack detail on the severity of cataracts or the potential for certain types of intraocular lenses to have blue-light blocking properties [40]. These factors are potentially important with regard to the development of SAD.

## 5. Conclusions

The significant number of participants in our study who were identified as being at risk of SAD/sub-SAD underscores the importance of screening for this disorder. Among patients with AMD and/or POAG, there is an increased risk of vision loss because of SAD or other types of depression [28,29,30]. Using the comprehensive VFQ-39 or expanding the VFQ-25 to include a subset of validated questions from the VFQ-39 could function to identify patients with vision loss who may suffer from SAD. This may reveal which patients are more likely to succeed with certain interventions, such as regularly taking eye drops for POAG or supplements to slow the progression of AMD. Our model may also be applicable to enhancing clinical trials aimed at assessing the vision-related quality of life from ophthalmic interventions.

## Figures and Tables

**Table 1:** Demographic and clinical characteristics.

Characteristics	All (111)	POAG (27) ^†^	AMD (70) ^†^	*p*-Value ^‡^
**Age (years)**				
Mean (SD)	81.1 (8.94)	80.6 (9.11)	80.9 (9.20)	0.891
Median	81.0	81.0	81.0	
Range	60–98	60–93	61–98	
**Visual acuity**				
Snellen range	20/20–LP	20/20–CF	20/20–LP	
Better eye, logMAR (SD)	0.390 (0.457)	0.178 (0.201)	0.437 (0.422)	<0.001
Worse eye, logMAR (SD)	0.969 (0.850)	0.537 (0.608)	1.058 (0.820)	0.003
Average, logMAR (SD)	0.680 (0.740)	0.357 (0.367)	0.748 (0.555)	0.001
**Optic nerve**				
Vertical CDR (SD)	0.480 (0.217)	0.720 (0.150)	0.364 (0.149)	<0.001
Range	0.10–0.95	0.30–0.95	0.10–0.90	
CDR difference (SD)	0.07 (0.09)	0.13 (0.11)	0.041 (0.06)	<0.001
**Race (%)**				0.108
White	99.1	96.3	100	
Other	0.90	3.7	0	
**Lens status (%)**				0.006
Bilateral pseudophakia	56.8	81.5	47.1	
Unilateral pseudophakia	18.0	11.1	15.7	
Phakic	25.2	7.41	37.1	
**Sex (%)**				0.755
Male	43.3	40.7	44.3	
Female	56.7	59.3	55.7	

**^†^** Excludes patients who had a diagnosis of both AMD and POAG, or other conditions that likely contributed to vision loss. **^‡^** Comparison between patients with only POAG or AMD. CF: count fingers, LP: light perception, SD: standard deviation.

**Table 2:** PCA model components by VFQ-39 question topics.

	Sub-Scale	Items
**Component 1:** Vision-associated problems ^†^	General vision	2, A2
	Distance activities	8, 9, 41, A6, A7, A8
	Near activities	5, 6, 7, A3, A4, A5
	Driving vision	15c, 16
	Color vision	12
	Peripheral vision	10
	Social functioning	11, 13, A9
**Component 2:** Quality of life ^†^	Role difficulties	17, 18, A11a, A11b
	Mental health	21, 22, 25, A12
	Dependency	20, 23, 24, A13
	Ocular pain	19

^†^ See Table S1 for details of the exploratory factor analysis statistics and composite scores for factors for PCA model component obtained from the VFQ-39 survey.

## Data Availability

Due to the nature of this research, participants of this study did not agree for their data to be shared publicly, so supporting data are not available.

## Supplementary Materials

The following supporting information can be downloaded at: https://www.mdpi.com/article/10.3390/vision6020032/s1, Figure S1: Area under receiver operating characteristics (ROC) curve for the final subscale model of questions on the VFQ-39 used to discriminate participants at risk of SAD/sub-SAD from those participants who did not screen positive based on their GSS score. Table S1: Primary Components Analysis (PCA) Statistics and Composite Scores for Factors in the NEI-VFQ-39 Questionnaire.
