# Supplementary material for: Vision-Related Quality of Life and Seasonal Affective Disorder in Patients with Glaucoma and Macular Degeneration
Source: Vision (Basel). 2022 Jun 14;6(2):32. doi: 10.3390/vision6020032 (PMC9227504; doi:10.3390/vision6020032)
Supplement: Supplementary file 1 [file vision-06-00032-s001.zip › vision-1727517-supplementary.pdf]

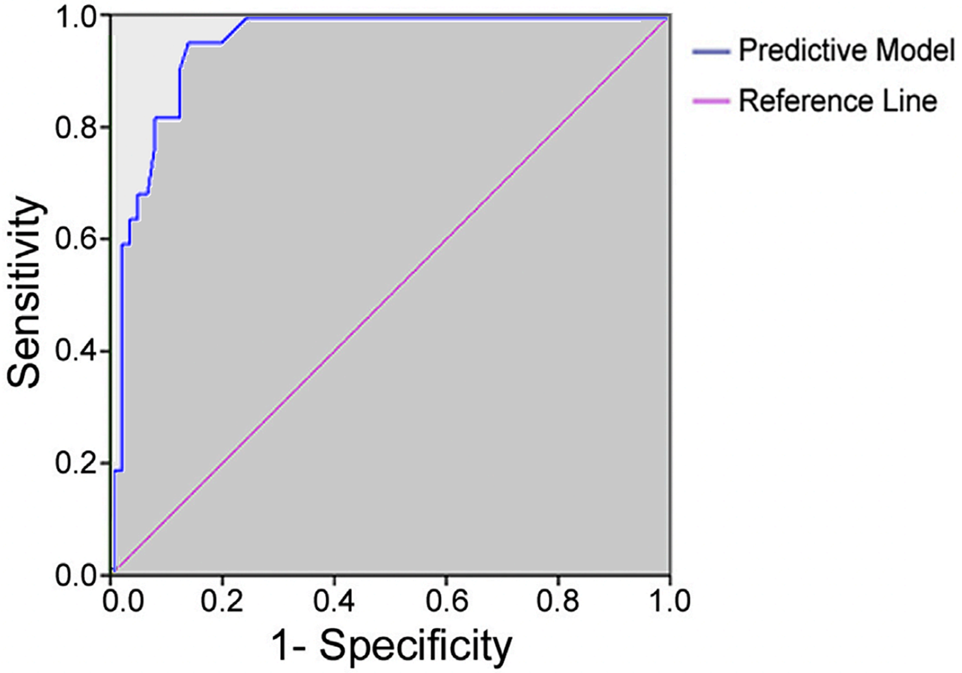

Figure S1. Area under receiver operating characteristic (ROC) curve for the final subscale model of questions on the VFQ-39 used to discriminate participants at risk of SAD/sub-SAD from those participants who did not screen positive based on their GSS score.

Table S1: Primary Components Analysis (PCA) Statistics and Composite Scores for Factors in the NEI-VFQ-39 Questionnaire		Communality	Component	
			Vision-associated problems	Quality of life
VF2	At the present time, would you say your eyesight using both eyes (with glasses or contact lenses, if you wear them) is excellent, good, fair, poor, or very poor or are you completely blind?	0.677	0.703	0.429
VFA2	How would you rate your eyesight now (with glasses or contact lens on, if you wear them), on a scale of from 0 to 10, where zero means the worst possible eyesight, as bad or worse than being blind, and 10 means the best possible eyesight?	0.469	0.58	0.365
VF8	How much difficulty do you have reading street signs or the names of stores?	0.567	0.598	0.457
VF9	Because of your eyesight, how much difficulty do you have going down steps, stairs, or curbs in dim light or at night?	0.491	0.612	0.342
VF14	Because of your eyesight, how much difficulty do you have going out to see movies, plays, or sports events?	0.595	0.683	0.358
VFA6	Because of your eyesight, how much difficulty do you have recognizing people you know from across a room?	0.564	0.603	0.448
VFA7	Because of your eyesight, how much difficulty do you have taking part in active sports or other outdoor activities that you enjoy (like golf, bowling, jogging, or walking)?	0.572	0.686	0.318
VFA8	Because of your eyesight, how much difficulty do you have seeing and enjoying programs on TV?	0.648	0.7	0.399
VF5	How much difficulty do you have reading ordinary print in newspapers?	0.556	0.649	0.367
VF6	How much difficulty do you have doing work or hobbies that require you to see well up close, such as cooking, sewing, fixing things around the house, or using hand tools?	0.622	0.678	0.402
VF7	Because of your eyesight, how much difficulty do you have finding something on a crowded shelf?	0.665	0.696	0.424
VFA3	Wearing glasses, how much difficulty do you have reading the small print in a telephone book, on a medicine bottle, or on legal forms?	0.557	0.626	0.406
VFA4	Because of your eyesight, how much difficulty do you have figuring out whether bills you receive are accurate?	0.631	0.723	0.33
VFA5	Because of your eyesight, how much difficulty do you have doing things like shaving, styling your hair, or putting on makeup?	0.614	0.658	0.425
VF15c	IF CURRENTLY DRIVING: How much difficulty do you have driving during the daytime in familiar places?	0.373	0.508	0.338
VF16	How much difficulty do you have driving at night?	0.084	0.228	0.179
VF12	Because of your eyesight, how much difficulty do you have picking out and matching your own clothes?	0.582	0.645	0.407
VF10	Because of your eyesight, how much difficulty do you have noticing objects off to the side while you are walking along?	0.507	0.651	0.287
VF11	Because of your eyesight, how much difficulty do you have seeing how people react to things you say?	0.528	0.626	0.369
VF13	Because of your eyesight, how much difficulty do you have visiting with people in their homes, at parties, or in restaurants?	0.497	0.599	0.373
VFA9	Because of your eyesight, how much difficulty do you have entertaining friends and family in your home?	0.405	0.578	0.267
VF17	Do you accomplish less than you would like because of your vision?	0.743	0.739	-0.443
VF18	Are you limited in how long you can work or do other activities because of your vision?	0.828	0.715	-0.563
VFA11a	Do you have more help from others because of your vision?	0.787	0.645	-0.61
VFA11b	Are you limited in the kinds of things you can do because of your vision?	0.812	0.707	-0.558
VF21	I feel frustrated a lot of the time because of my eyesight.	0.651	0.65	-0.478
VF22	I have much less control over what I do, because of my eyesight.	0.78	0.778	-0.419
VF25	I worry about doing things that will embarrass myself or others, because of my eyesight.	0.803	0.516	-0.733
VFA12	I am often irritable because of my eyesight.	0.768	0.51	-0.713
VF20	I stay home most of the time because of my eyesight.	0.867	0.708	-0.605
VF23	Because of my eyesight, I have to rely too much on what other people tell me.	0.877	0.755	-0.554
VF24	I need a lot of help from others because of my eyesight.	0.89	0.75	-0.572
VFA13	I don’t go out of my home alone, because of my eyesight.	0.796	0.661	-0.598
VF19	How much does pain or discomfort in or around your eyes, for example, burning, itching, or aching, keep you from doing what you’d like to be doing?	0.69	0.393	-0.732
